# Lead toxicosis of captive vultures: case description and responses to chelation therapy

**DOI:** 10.1186/1746-6148-9-11

**Published:** 2013-01-16

**Authors:** Jiri Pikula, Pavlina Hajkova, Hana Bandouchova, Ivana Bednarova, Vojtech Adam, Miroslava Beklova, Jiri Kral, Karel Ondracek, Jitka Osickova, Miroslav Pohanka, Jana Sedlackova, Hana Skochova, Jakub Sobotka, Frantisek Treml, Rene Kizek

**Affiliations:** 1 Department of Veterinary Ecology and Environmental Protection, Faculty of Veterinary Hygiene and Ecology, University of Veterinary and Pharmaceutical Sciences Brno, Brno, Czech Republic; 2 Avian Veterinary Clinic Skrivany, Cesky Brod, Czech Republic; 3 Department of Chemistry and Biochemistry, Mendel University, Brno, Czech Republic; 4 Centre of Advanced Studies, Faculty of Military Health Sciences, University of Defence, Hradec Kralove, Czech Republic; 5 Department of Infectious Diseases and Microbiology, Faculty of Veterinary Medicine, University of Veterinary and Pharmaceutical Sciences Brno, Brno, Czech Republic

**Keywords:** *Aegypius monachus*, *Neophron percnopterus*, Plumbism, Haematology, Biochemistry, Oxidative stress, Reproduction impairment, Treatment

## Abstract

**Background:**

Lead, a serious threat for raptors, can hamper the success of their conservation. This study reports on experience with accidental lead intoxication and responses to chelation therapy in captive Cinereous (*Aegypius monachus*) and Egyptian (*Neophron percnopterus*) Vultures.

**Results:**

Soil contamination by lead-based paint sanded off the steel aviary resulted in poisoning of eight Cinereous and two Egyptian Vultures. A male Egyptian Vulture developed signs of apathy, polydipsia, polyuria, regurgitation, and stupor, and died on the next day. Liver, kidney and blood lead concentrations were 12.2, 8.16 and 2.66 μg/g, respectively. Laboratory analyses confirmed severe liver and kidney damage and anaemia. Blood Pb levels of Pb-exposed Cinereous Vultures were 1.571 ± 0.510 μg/g shortly after intoxication, decreased to 0.530 ± 0.165 μg/g without any therapy in a month and to 0.254 ± 0.097 μg/g one month after CaNa_2_EDTA administration. Eight months later, blood lead levels decreased to close to the background of the control group. Blood parameters of healthy Pb-non-exposed Cinereous Vultures were compared with those of the exposed group prior to and after chelation therapy. Iron levels in the lead-exposed pre-treatment birds significantly decreased after chelation. Haematocrit levels in Pb-exposed birds were significantly lower than those of the controls and improved one month after chelation. Creatine kinase was higher in pre-treatment birds than in the controls but normalised after therapy. Alkaline phosphatase increased after chelation. A marked increase in the level of lipid peroxidation measured as thiobarbituric acid reactive species was demonstrated in birds both prior to and after chelation. The ferric reducing antioxidant power was significantly lower in pre-treatment vultures and returned to normal following chelation therapy. Blood metallothionein levels in lead-exposed birds were higher than in controls. Reduced glutathione dropped after CaNa_2_EDTA therapy, while oxidised glutathione was significantly lower in both pre- and post-treatment birds. A chick in an egg produced by a Cinereous Vulture female two months after lead toxicosis died on day 40 of artificial incubation. Lead concentrations in foetal tissues were consistent with levels causing avian mortality.

**Conclusions:**

The reported blood parameters and reproduction impairment in captive birds may have implications for professionals dealing with lead exposure in wild birds.

## Background

Lead toxicosis is the most common form of heavy metal toxicity in birds and is now probably the most frequent poisoning induced by a contaminant in avian species worldwide [1]. Both free-living and captive birds are at risk from lead poisoning. Aquatic and terrestrial birds can be exposed to lead through the ingestion of ammunition sources such as shots or of fishing sinkers [2], while local sediment contamination represents another significant hazard for birds, for example, geese, swans and diving ducks [3-6].

Victims of ingestion of lead ammunition are often raptors feeding upon dead or injured game animals [2,7,8]. Lead poisoning has been reported for many raptor species, which is of conservation concern [8-13]. This is also the case for the globally threatened Cinereous Vulture (*Aegypius monachus*) population [14].

Lead, when absorbed in a sufficient quantity, can exert negative effects at various levels of organisation, from biochemical disruption to reproductive impairment [15,16]. While both avian genders remove heavy metals through excretion and deposition in feathers [17,18], females can also eliminate heavy metals in the contents of their eggs [18]. This excretion route, however, endangers the avian embryo.

The present paper is a case report on experience with lead intoxication in a group of two Egyptian Vultures (*Neophron percnopterus*) and eight Cinereous Vultures (*Aegypius monachus*) housed at the Zoo of the Capital City of Prague, Czech Republic, in 2009. The effects of lead and chelation therapy on toxicological, biochemical, and oxidative stress parameters were evaluated and the results obtained from poisoned birds were compared with those from non-exposed vultures. Concentrations of lead in tissues of a foetus dying on day 40 of incubation and the eggshell of the egg laid by a Cinereous Vulture female two months after lead exposure were also measured and discussed.

## Methods

### Captive birds, case history and samples collected

A total of eight captive Cinereous Vultures and two Egyptian Vultures were accidentally exposed to lead contamination in their aviary. The characteristics of these birds were as follows: five females and five males, age ranging from 8 to 36 years, body weight from 7 to 11.5kg (*Aegypius monachus*) and from 1.6 to 2.2kg (*Neophron percnopterus*). Both vulture species were kept together in a large exhibit at the Zoo of the Capital City of Prague, Czech Republic. The steel wires of the aviary were repainted in 2008 after the old minium paint (lead tetroxide) had been removed mechanically. Unfortunately, less attention was paid to the decontamination of the soil polluted by paint debris.

Adult birds not exposed to lead kept at the Zoo Liberec (Czech Republic) were used as healthy controls (i.e. six Cinereous Vultures, gender ratio 1:1). Blood samples were collected only once from Pb-non-exposed control Cinereous Vultures, while the blood of lead-affected birds was sampled for haematology, biochemistry and toxicology analyses shortly after exposure (at the time of diagnosis), 1 (pre-treatment birds), 2 (post-treatment birds), and 8 months after lead exposure. The pre- and post-treatment vultures were also sampled at the time of CaNa_2_EDTA therapy and one month after. Ten mixed soil samples were collected for heavy metal analysis at different sites in the aviary from the surface layer to a depth of 10 cm.

### Chelation therapy

The Egyptian Vulture male died shortly after the onset of clinical signs; therefore, trial chelation therapy was started with oral D-penicillamine in the Egyptian Vulture female (50mg/kg twice daily, Metalcaptase 300 Por Tbl Flm, Heyl Chemisch-pharmazeutische Fabrik GmbH & Co. KG, Berlin, Germany). This treatment, however, had to be discontinued after three days because of adverse effects in this bird. As a suitable CaNa_2_EDTA (calcium disodium ethylenediaminetetraacetate) injection product had been unavailable at that time, it was only possible to continue with chelation therapy 1 month after lead exposure (30mg/kg for 5 days, subcutaneous application, EDTA-Calcium Dinatrium, 1g/10ml ampoule, Sopharma, Bulgaria). The surviving female Egyptian Vulture and eight Cinereous Vultures were subjected to this CaNa_2_EDTA therapy.

### Examination of birds that died

Apart from blood sampling, the male Egyptian Vulture was radiographed (POSKOM PXP-40HF diagnostic x-ray unit, Poskom Co., Ltd., Korea; 5mAs and 50kV exposure) and subjected to necropsy, histopathology and toxicological analyses for heavy metals (lead) in tissues such as the liver, kidney, and blood. Samples of liver and kidney tissue were collected into 10% buffered formalin, treated using a routine histological technique and embedded in paraffin. 5μm sections of the paraffin blocks were made and stained with haematoxylin and eosin. A male Cinereous Vulture died approximately 14 months after exposure due to a cause unrelated to lead toxicity and its liver, kidney and bone (femur) samples were also examined for lead concentration.

### Egg and foetus analysis

A Cinereous Vulture female laid a normal-sized egg two months after exposure to lead. The egg was artificially incubated and, after development ceased, was dissected to measure lead concentrations in the yolk, albumen, liver, bone (femur), kidney, central nervous system, and eggshell. The total lead content was estimated using weights and lead concentrations measured in the above-mentioned individual egg and foetus components.

### Plasma biochemistry and haematology

Blood (2ml) was collected from the right jugular vein using a 2ml heparinised syringe (B.Brown Injekt^(R)^, Germany) and a 25 gauge needle (0.5x25mm, Terumo Europe, Belgium), and was processed as previously described [19,20]. Blood samples were divided equally for whole blood analyses and centrifugation to obtain plasma. Whole blood samples were analysed using an automated analyser (SPOTCHEM^TM^ EZ SP-4430, ARKRAY, Japan) for calcium (mmol/l), phosphorus (mmol/l), haemoglobin (g/l), total protein (g/l), uric acid (mmol/l), aspartate aminotransferase (μkat/l), alkaline phosphatase (μkat/l), creatine kinase (μkat/l), lactate dehydrogenase (μkat/l), and bilirubin (μmol/l). Haematocrit (l/l) was measured using microhaematocrit heparinised centrifuge capillary tubes.

### Antioxidant parameters

Lipid peroxidation was analysed as previously described [21,22]. Briefly, the level of lipid peroxidation in avian plasma samples was assessed as total thiobarbituric acid reactive species (TBARS). The ferric reducing ability of plasma (FRAP assay) was performed according to standard methods [23] with minor modifications [24,25]. Blood plasma samples collected from vultures were also examined for the content of metallothionein (MT), reduced glutathione and oxidised glutathione. MT was determined using the differential pulse voltammetry Brdicka reaction as previously described [26-28]. Reduced and oxidised glutathione forms were assayed by high-performance liquid chromatography with electrochemical detection [29].

### Element analysis

Element analysis of whole blood, tissue and soil samples was performed by inductively-coupled plasma–mass spectrometry (ICP-MS; Agilent 7500ce, Agilent Technologies, Japan) as described in our previous article [20].

An electrochemical analyser (Metrohm AG, Switzerland) was also used for determination of Pb(II) [27]. The analyser (797 VA Computrace from Metrohm, Herisau, Switzerland) employs a conventional three-electrode configuration with a hanging mercury drop working electrode, Ag/AgCl/3MKCl as a reference electrode, and a platinum auxiliary electrode. Differential pulse voltammetric measurements were carried out under the following parameters: deoxygenation with argon 60s, deposition potential -1.3V, time of deposition 240s, start potential -1.3V, end potential 0.15V, pulse amplitude 0.025V, pulse time 0.04s, step potential 5.035mV, time of step potential 0.3s.

### Statistical analysis

The data analysis software Statistica for Windows® 10 (StatSoft, Inc., Tulsa, OK, USA) was used to compare different groups of vultures by one-way analysis of variance (ANOVA) and post-hoc analysis of means by the LSD test. Levene’s method was used to test for the homogeneity of variances. Non-homogenous parameters were log-transformed prior to analysis and compared with the non-parametric Kruskal-Wallis test. All tests were considered statistically significant and highly significant when resulting in values of p <0.05 and p <0.01, respectively.

## Results

### Clinical description of the case

**Figure 1:**
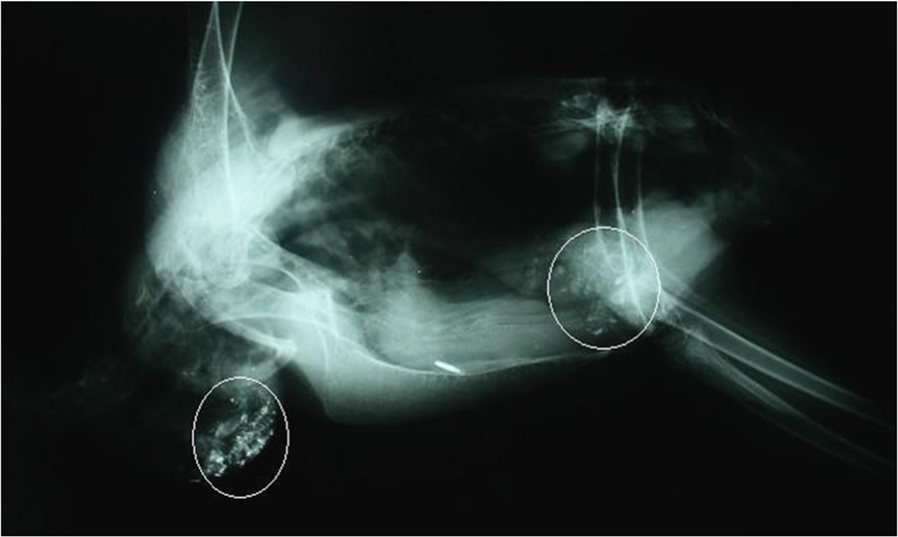
**Radiographic evidence of lead particles in the digestive tract.** Radiopaque material present within the crop and stomach (highlighted by circles) of a male Egyptian Vulture (*Neophron percnopterus*), lateral projection.

A male Egyptian Vulture developed clinical signs of apathy, polydipsia, polyuria, regurgitation, and stupor. Foul-smelling fluid was flowing freely from its beak, there was crop distension due to food stagnation and it died on the next day. The suspected lead toxicosis was confirmed radiographically as there were many small radiopaque chips in the crop and stomach (cf. Figure 1). The soil surface layer examination yielded levels of 1.00 to 25.85 mg/g of lead, and liver, kidney and blood lead concentrations were 12.2, 8.16 and 2.66 μg/g, respectively. Laboratory examination showed marked deterioration of haematology and biochemistry values in this bird: haemoglobin = 71 g/l, haematocrit = 0.28 l/l, calcium = 3.41 mmol/l, phosphorus = 7.68 mmol/l, uric acid = 11,71 μmol/l, aspartate aminotransferase = 26.92 μkat/l, alkaline phosphatase = 5.59 μkat/l, creatine kinase = 326.04 μkat/l, lactate dehydrogenase = 29 μkat/l. Gross lesions included swollen pale kidneys and liver, dilated gallbladder and blood-tinged intestinal contents. Histopathology revealed renal tubular necrosis, marked perivascular infiltration by mononuclear cells and pericentral distribution of granular dystrophy and hepatocellular necrosis in the liver. No other vultures died or showed clinical signs of lead intoxication at that time. The blood lead levels decreased from 1.07 μg/g to 0.28 μg/g within two months in the cage-mate female Egyptian Vulture. First, this female was treated with D-penicillamine, but because of adverse effects, such as inappetence and regurgitation, this was changed for CaNa_2_EDTA a month later.

### Development of blood lead levels in Cinereous Vultures

**Figure 2:**
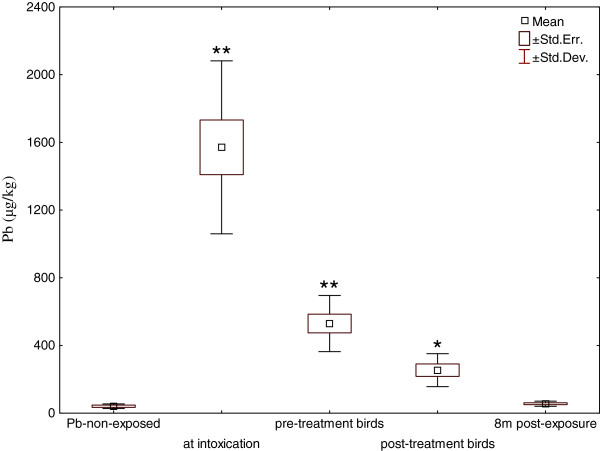
**Development of blood lead levels in Cinereous Vultures *(Aegypius monachus).*** N = 6 in healthy Pb-non-exposed controls, n = 8 in birds sampled at the time of diagnosis, at the time of and one month after CaNa_2_EDTA injection (pre-and-post-treatment birds, respectively) and 8 months after exposure to lead; * = p < 0.05, ** = p < 0.01 when compared against the healthy control group.

As shown in Figure 2, background blood lead concentration in control healthy Pb-non-exposed birds was 0.041 ± 0.013 μg/g and blood analyses of eight Cinereous Vultures accidentally exposed to Pb resulted in values of 1.571 ± 0.510 μg/g shortly after exposure (i.e. at the time of diagnosis) that decreased within a month to 0.530 ± 0.165 μg/g without any therapy (pre-treatment birds) and to 0.254 ± 0.097 μg/g (post-treatment birds) one month after CaNa_2_EDTA administration. Eight months later, the blood lead levels of Pb-exposed birds decreased close to the control group (0.056 ± 0.015 μg/g). Examination of the Cinereous Vulture male that died of causes unrelated to the lead exposure provided data on concentrations of lead approximately 14 months after exposure in liver, kidney, and bone (femur) amounting to 0.01, 0.12, and 4.44 μg/g, respectively.

### Biochemistry and antioxidant parameters in Cinereous Vultures

Table 1 presents selected blood parameters of healthy Pb-non-exposed birds and Cinereous Vultures prior to and after therapy for lead toxicosis. There were no statistically significant differences in calcium and phosphorus levels. However, therapy with CaNa_2_EDTA resulted in a decrease of both. Iron levels were lower, but not significantly, in the lead-exposed pre-treatment birds and decreased significantly following chelation therapy. Decreased haemoglobin and haematocrit levels of Pb-exposed birds improved one month after CaNa_2_EDTA administration. There were no statistically significant differences in total protein levels in the three groups of vultures. Both uric acid and aspartate aminotransferase showed greater levels in pre-treatment birds that normalised after therapy. A similar trend was detected with creatine kinase activity. In comparison with healthy controls, alkaline phosphatase was not statistically different prior to but increased after therapy with CaNa_2_EDTA. No statistical differences were observed in lactate dehydrogenase activity. When compared with controls, total bilirubin was lower both in pre- and post-treatment vultures, but the differences were not significant. A marked increase in the level of lipid peroxidation measured as the total thiobarbituric acid reactive species was demonstrated both in birds prior to and after treatment for lead toxicosis. The ferric reducing antioxidant power was significantly lower in the pre-treatment vultures and returned to normal following chelation therapy. Blood metallothionein levels in lead-exposed birds were higher than in control vultures, but only the high levels in the post-treatment birds were significantly different from those of the controls. As shown in comparison with healthy controls, reduced glutathione dropped after CaNa_2_EDTA therapy, while oxidised glutathione had a decreasing tendency both in pre-treatment and post-treatment birds.

**Table 1:** **Blood parameters of healthy Pb-non-exposed birds and Cinereous Vultures *(Aegypius monachus ) *prior to and after therapy for lead toxicosis using CaNa_2_EDTA**

**Parameters**	** Groups of vultures**		
	**Pb-non-exposed**	**Pre-treatment**	**Post-treatment**
Ca (mmol/l)	2.83 ± 0.35	2.74 ± 0.25	2.50 ± 0.35
P (mmol/l)	1.28 ± 0.15	1.30 ± 0.27	0.96 ± 0.35
Fe (μmol/l)	13.55 ± 3.27	10.57 ± 1.74	5.40 ± 2.65**
Hb (g/l)	147.00 ± 27.12	112.12 ± 19.85	132.71 ± 24.06
Ht (l/l)	0.50 ± 0.04	0.36 ± 0.06*	0.42 ± 0.08
TP (g/l)	35.51 ± 6.40	30.80 ± 5.49	32.21 ± 4.82
UAC (μmol/l)	578.85 ± 202.97	872.30 ± 35.43	731.85 ± 327.45
AST (μkat/l)	6.78 ± 1.54	7.81 ± 1.90	5.74 ± 2.77
ALP (μkat/l)	0.64 ± 0.21	1.00 ± 1.62	3.71 ± 1.34*
CK (μkat/l)	13.53 ± 6.58	41.04 ± 10.54*	10.63 ± 0.94
LDH (μkat/l)	7.82 ± 0.94	8.06 ± 4.81	13.17 ± 8.62
TBIL (μmol/l)	13.10 ± 2.29	10.60 ± 1.50	7.81 ± 2.48
TBARS (nmol/l)	1.94 ± 0.12	7.00 ± 0.80**	7.31 ± 0.45**
FRAP (μmol/l)	1619.41 ± 34.00	1046.22 ± 362.14**	1516.75 ± 451.43
MT (μg/mg protein)	1.43 ± 0.16	1.79 ± 0.40	1.92 ± 0.20*
GSH (μg/ml)	1.00 ± 0.05	1.07 ± 0.12	0.79 ± 0.03*
GSSG (μg/ml)	0.11 ± 0.04	0.05 ± 0.01*	0.01 ± 0.001**

Values represent mean ± SD; n = 6 in healthy Pb-non-exposed birds (3 males and 3 females), n = 8 in pre-and-post-treatment birds; * = p < 0.05, ** = p < 0.01 when compared against control group. The pre-and-post-treatment birds were blood sampled at the time of and one month after Ca-EDTA therapy, respectively. Ca = calcium, P = phosphorus, Fe = iron, Hb = haemoglobin, Ht = haematocrit, TP = total protein, UAC = uric acid, AST = aspartate aminotransferase, ALP = alkaline phosphatase, CK = creatine kinase, LDH = lactate dehydrogenase, TBIL = total bilirubin, TBARS = thiobarbituric acid reactive species, FRAP = ferric reducing ability of plasma, MT = metallothionein, GSH = reduced glutathione, GSSG = oxidised glutathione.

### Egg and foetus analysis

The egg produced by a Cinereous Vulture female two months after lead intoxication was artificially incubated and ceased to develop on day 40 of incubation. Analysis of the egg and foetus components for lead revealed the following concentrations: yolk 2.48, albumen 2.60, liver 8.00, bone (femur) 0.66, kidney 7.45, central nervous system 3.32, and eggshell 2.14 (μg Pb/g). The total lead content estimate for this 229g egg amounted to 1.18mg.

## Discussion

Clinical lead toxicosis occurred one month after the captive vultures were returned to their aviary following old minium paint removal. Although ten birds had been exposed to severe lead contamination, only one died. The vultures were poisoned with lead(II) ions in the paint dust and chips that were sanded off the steel aviary construction and contaminated the soil to such an extent that its 10cm-deep surface layer contained as much as 25.85 mg/g of lead. Vultures are scavengers feeding on meat on the bone and entire carcasses or parts thereof supplied on the ground. Therefore, feeding on soiled carrion from the ground was the direct way of lead exposure for these captive vultures. Generally, kidney and liver concentrations of lead above 8-10 μg/g on a fresh weight basis are diagnostic for lead toxicosis in animals [30]. Liver, kidney and blood lead concentrations in the male Egyptian Vulture that died were consistent with acute intoxication and levels causing mortality [1]. Likewise, laboratory analyses confirmed severe liver and kidney damage and anaemia [31]. The present case is similar to an outbreak of lead toxicosis in captive Houbara Bustards (*Chlamydotis undulata macqueenii*) on a private farm in the United Arab Emirates where the birds ingested large flakes of green paint peeling off the metal poles supporting the aviary mesh [32]. Fortunately, the blood lead concentration was approximately 2.5 times lower in the cage-mate female Egyptian Vulture and decreased with D-penicillamine and later CaNa_2_EDTA treatment.

Background blood lead concentrations in Pb-non-exposed control Cinereous Vultures were in agreement with those measured in other birds [17,20,33,34]. Regarding the development of blood lead levels in Cinereous Vultures surviving the lead toxicosis, it is important to realise that initially high sub-lethal values decreased even without therapy within the first month after lead ingestion, probably due to excretion and deposition in bones [18] and that the decrease of blood lead levels following CaNa_2_EDTA administration was not as substantial as might be expected (cf. Figure 2).

Interestingly, it was possible to examine the temporal development of the intoxication and evaluate both adverse effects of lead and effects of chelation therapy on selected blood parameters in the group of surviving Cinereous Vultures.

Calcium levels are reported not to change in lead-exposed falcons, while phosphorus rises [35]. Unlike Wernery et al. [35], this study found phosphorus levels equal and lower in pre-treatment and post-treatment Cinereous Vultures, respectively, with the exception of the extreme of phosphorus rise in the male Egyptian Vulture who died. The current data also document a considerable decrease of iron both in pre-treatment and post-treatment birds. This iron decrease in lead-exposed birds is related to the development of anaemia, whereas it is the effect of CaNa_2_EDTA inducing urinary excretion of iron in the post-treatment birds [36]. Importantly, iron deficiency is known to increase susceptibility to lead poisoning in infants and children [37].

Chelation therapy effectively improved haematocrit in Pb-exposed vultures in the present study. This parameter reflects the extent and efficiency of oxygen-carrying capacity of blood and thus the bird’s ability for physical performance and is often used as an indicator of condition in birds [31]. Blood collection is considered non-destructive sampling for the lead toxicosis diagnostics and clinical state of health evaluation [33]; however, stress of handling can result in death in anaemic birds.

There were no significant differences in total protein levels of control healthy Pb-non-exposed birds and pre- and post-treatment vultures, and the values were within published normal ranges of the Cinereous Vulture [31].

The significant increase of alkaline phosphatase activity in the post-treatment group of vultures may be explained by collagen degradation and induction of bone metabolism after CaNa_2_EDTA therapy [38]. While the activity of this enzyme is a measure of condition in nestlings and growing birds [31], it may be hypothesised that chelation therapy-induced bone resorption may rather result in poor performance.

The significant increase of creatine kinase activity in lead-exposed vultures well corresponds with published data [35] and a return to normal of this enzyme activity provides information on the efficacy of chelation therapy. Unfortunately, normal plasma chemistry values of enzymes were published in concentration units [31] and do not allow for a comparison with activities of the current study.

Lead impairs general health, growth, immune, reproductive, and many other physiological functions in birds, the biosynthesis of haemoglobin inclusive [39]. Some of its adverse effects are associated with the oxidative damage of lipids, proteins, and DNA [40]. The current results on lipid peroxidation (TBARS) and the ferric reducing ability of plasma (FRAP assay) demonstrate oxidative stress in lead-exposed vultures (cf. Table 1). While the FRAP values returned to normal following therapy with CaNa_2_EDTA, TBARS slightly increased in comparison with pre-treatment birds. Data on the increase of TBARS correspond with published findings that demonstrate enhanced lipid peroxidation in the liver of rats treated with CaNa_2_EDTA [36].

The total antioxidant capacity of blood can be measured using the ferric reducing antioxidant power assay as a clinical marker of oxidative stress. The value of this parameter depends on non-enzymatic antioxidants including ascorbic and uric acids, bilirubin, vitamin E, α-tocopherol and albumin and is linearly related to their molar concentrations. Uric acid contributes around 60% to the plasma value [23]. Uric acid, formed in the liver and eliminated by kidneys, represents the primary route of excretion of nitrogenous waste in birds [41].

Changes in the oxidation-reduction system of glutathione have also been demonstrated in the lead-exposed Cinereous Vultures both prior to and after CaNa_2_EDTA therapy. This phenomenon can be associated with the altered redox status and other damaged biochemical pathways due to the presence of lead(II) ions. Moreover, interactions of reduced glutathione with CaNa_2_EDTA can be considered due to the high reactivity of the –SH moiety of this abundant peptide. Considering the fact that over 90% of the total glutathione pool in healthy organisms is represented by its reduced form and an increased oxidised-to-reduced ratio is indicative of oxidative stress, both pre- and post-treatment birds were able to handle this issue and modulate the antioxidant response. A different response to lead exposure was reported from waterbirds [33] in which the level of oxidised glutathione in red blood cells increased in association with Pb levels >20 μg/dl.

On the other hand, inducible metallothioneins are the first detectable signs of exposure to heavy metals at the cellular level and may be used as biomarkers because there are probably constant levels of metallothioneins in non-stressed cells [42-45]. Metallothioneins binding various metal ions play a key role in reducing toxic effects of heavy metals in the organism [43].

Many environmental stressors can decrease immunocompetence in birds. Immunosuppressive effects, demonstrated even at low doses [46], can result in mortality because of other factors such as, for example, *Aspergillus* infection [5,47]. Combinations of several stressors, though sub-lethal when considered as a single exposure, are known to enhance avian toxicity [20,22].

Gross and microscopic lesions in the liver and kidney of the male Egyptian Vulture were consistent with lead toxicity [48]. The liver is important for the metabolism and excretion of lead in bile. Lead is characteristic for its strong affinity for the mitochondrial membrane and impairment of respiratory and phosphorylative abilities [49]. Importantly, there are common molecular mechanisms, such as oxidative stress and mitochondrial impairment, involved in the adverse effects of some toxins [40,50,51], as well as in the immune response [52].

Although all Cinereous Vultures survived a month after exposure and blood lead levels were decreasing, chelation therapy to reduce the risk of morbidity and mortality is considered in birds of prey such as California condors with blood lead levels ≥0.3-0.45 μg/g [17,53]. Such high levels of lead were present at the time of intoxication/diagnosis and in vultures prior to therapy. Treatment should continue until blood lead levels are below 0.2 μg/g [12]. As the blood lead concentrations were just around this threshold in the post-treatment birds (i.e. 0.254 ± 0.097 μg/g), the chelation therapy should probably have been more aggressive. What makes the decision to treat or not to treat the birds difficult is the lag between blood collection and availability of results of laboratory analyses for lead. It is imperative to minimize stressful stimuli to anaemic birds, therefore, one should both collect blood and administer chelation agents at one occasion.

It is also necessary to report some adverse effects such as inappetence and regurgitation after three days of D-penicillamine therapy in the Egyptian Vulture female. Side-effects of D-penicillamine are known to include gastrointestinal disturbances that may affect up to 30% of patients [54]. Importantly, vultures dosed for 5 days in the present study as well as birds treated with CaNa_2_EDTA for as many as 23 consecutive days, did not demonstrate any deleterious effects [55]. While D-penicillamine was administered *per os*, CaNa_2_EDTA is a parenteral agent chelated with Ca^2+^ and thus selective for divalent cations with greater affinity for EDTA, which it will exchange for Ca^2+^.

As there are no veterinary chelators, the use of both human drugs containing D-penicillamine and CaNa_2_EDTA was extra-label. In light of this, no standard protocol is available for birds and, for example, California condors are considered in need of chelation therapy based on a threshold level recommended for lead-poisoned children [53]. Importantly, one must bear in mind the possible harmful effects of chelation therapy. Nephrotoxicity of CaNa_2_EDTA can further deteriorate the renal function impaired by exposure to lead [56]. While heavy metals including lead are well known embryo/foetal toxicants, the teratogenic potential of chelating agents has also been demonstrated to be due to depletion of essential trace elements such as copper or zinc induced by chelation [57]. Parenteral administration of CaNa_2_EDTA can be painful, it may result in muscle damage at the injection site [56] and hamper early release of birds from captive facilities. For example, semiannual recapture of California condors for health checkups indicated the need of clinical intervention to avert morbidity and mortality in about 20% of birds [53]. However, extensive stays in captivity associated with chelation therapy can probably result in behavioural and reproductive problems due to disruption of pair bonds and inability to maintain nesting territories [58]. Chelating agents reduce body stores of lead. On the other hand, the reduction of blood lead levels may result in the mobilisation of skeletal stores of lead associated with redistribution of lead concentrations and worsening signs of toxicity. Multiple treatments may, therefore, be indicated by follow-up tests [59]. As shown by the present pre- and post-treatment data in lead-poisoned vultures, the benefits and risks of chelation therapy should carefully be considered in individual birds because only some blood parameters normalised after CaNa_2_EDTA administration (i.e., haematocrit, creatine kinase and ferric reducing ability of plasma) and others worsened. Nevertheless, it is these authors’ opinion that chronic sublethal effects of lead exposure associated even with lower blood lead levels outweigh the adverse action of chelation therapy in decision-making how to manage these cases because there is no apparent threshold below which adverse effects of lead do not occur [60]. Apart from measures to decrease the lead burden, supportive therapy is often necessary in poisoned birds. Taken together, these results warrant supplying lead-intoxicated and CaNa_2_EDTA-treated birds with antioxidants, iron and calcium to counteract the adverse effects both of lead exposure and chelation therapy [54,56].

There is a diversity of environmental pollutants that can impair avian reproduction either through the action on adult birds and developing embryos and foetuses, or both [61-63]. Lead, affecting multiple physiological functions in birds [39], belongs to such toxins. Its accumulation can exert reproductive effects in breeding birds and detrimental effects may include changes in egg size, eggshell thickness, hatchability and fledging success [16]. The reproductive success was found to be negatively correlated with maternal Pb [64]. Female birds are known to eliminate heavy metals in their eggs [18]. While blood lead levels reflect recent exposure, egg contamination can be due to recent or chronic exposure [8,9] and result both from maternal blood and lead mobilisation from organs and tissues during egg formation [64]. The clutch of the Cinereous Vulture typically includes only a single egg of approximately 237g [65]. The Cinereous Vulture female in this study laid a normal sized egg. Regarding the small clutch size and the total lead content estimate of 1.18mg, this excretion route cannot represent an effective way of depuration in this species. However, as shown by the lead concentration in the foetal liver consistent with levels causing mortality in birds [1], it can pose a threat for the developing avian egg. Incubation lasts from 52 to 55 days in the Cinereous Vulture and, despite the high Pb burden in the egg, the development ceased not earlier than on day 40. In agreement with published data [18], lead concentrations were higher in the contents compared to the eggshell. Reproduction during subsequent breeding seasons can also be at risk from lead mobilised from bone deposits because birds that have survived acute exposure may have relatively high bone Pb. Interestingly, the femur Pb concentration found in the Cinereous Vulture male 14 months after exposure was lower than that indicative of excessive exposure and observed in birds that have died of Pb poisoning [8,9]. This bird’s lead burden was probably further decreased by chelation therapy.

Bone lead concentrations in long-lived species of birds reflect life time exposure and increase with age [9]. Although this bioaccumulation is less-readily related to pathophysiological mechanisms and mortality when compared with soft tissues and blood levels [8], sub-lethal effects can result in bone composition alteration such as lower degree of mineralisation and higher bone fragility [9].

The global populations of both avian scavengers are declining and their conservation status is near threatened and endangered in the Cinereous Vulture (*Aegypius monachus*) and Egyptian Vulture (*Neophron percnopterus*), respectively [66]. Lead exposure of captive birds with its lifelong health consequences and reproduction impairment can hamper the success of conservation breeding and make birds unavailable for reintroductions [8,67]. In view of this, the accident was a serious management failure. As the danger of lead is notoriously known, the decision not to remediate the soil prior to restocking birds in the aviary is hard to understand.

## Conclusions

These results document toxicokinetics and toxicodynamics of lead and chelation therapy in two species of vultures. While the study contributes to the understanding of the pathogenic mechanisms of lead exposure in birds, knowledge on the development of blood parameters may also prove useful as future reference in the clinical evaluation of vultures. The reported data on captive birds may thus have implications for wildlife biologists, veterinarians and conservationists of avian biodiversity dealing with lead exposure in wild birds of prey.

## Competing interests

The authors declare that they have no competing interests.

## Authors’ contributions

JP supervised the whole study and performed data analyses. PH diagnosed the toxicosis in vultures, treated and sampled the birds. HB, MB and FT drafted the manuscript. JK, KO, JO performed biochemical evaluations. SJ, HS measured haematological parameters. MP and JS analysed blood samples for the total antioxidant capacity and lipid peroxidation. IB measured parameters in the avian egg contents and the foetus. VA and RK analysed metallothioneins, glutathione and heavy metals. All authors read and approved the final manuscript.
